# Unconventional excited-state dynamics in the concerted benzyl (C_7_H_7_) radical self-reaction to anthracene (C_14_H_10_)

**DOI:** 10.1038/s41467-022-28466-7

**Published:** 2022-02-10

**Authors:** Ralf. I. Kaiser, Long Zhao, Wenchao Lu, Musahid Ahmed, Vladislav S. Krasnoukhov, Valeriy N. Azyazov, Alexander M. Mebel

**Affiliations:** 1 Department of Chemistry, University of Hawaii at Manoa, Honolulu, HI 96822 USA; 2 Chemical Sciences Division, Lawrence Berkeley National Laboratory, Berkeley, CA 94720 USA; 3 Samara National Research University, Samara 443086 and Lebedev Physical Institute, 443011 Samara, Russian Federation; 4 Department of Chemistry and Biochemistry, Florida International University, Miami, FL 33199 USA

**Keywords:** Reaction kinetics and dynamics, Excited states, Computational chemistry, Reaction mechanisms

## Abstract

Polycyclic aromatic hydrocarbons (PAHs) are prevalent in deep space and on Earth as products in combustion processes bearing direct relevance to energy efficiency and environmental remediation. Reactions between hydrocarbon radicals in particular have been invoked as critical molecular mass growth processes toward cyclization leading to these PAHs. However, the mechanism of the formation of PAHs through radical – radical reactions are largely elusive. Here, we report on a combined computational and experimental study of the benzyl (C_7_H_7_) radical self-reaction to phenanthrene and anthracene (C_14_H_10_) through unconventional, isomer-selective excited state dynamics. Whereas phenanthrene formation is initiated via a barrierless recombination of two benzyl radicals on the singlet ground state surface, formation of anthracene commences through an exotic transition state on the excited state triplet surface through cycloaddition. Our findings challenge conventional wisdom that PAH formation via radical-radical reactions solely operates on electronic ground state surfaces and open up a previously overlooked avenue for a more “rapid” synthesis of aromatic, multi-ringed structures via excited state dynamics in the gas phase.

## Abstract (web summary)

The reaction of benzyl radical self-reaction to anthracene opens-up a previously overlooked avenue for a more efficient synthesis of aromatic, multi-ringed structures via excited state dynamics in the gas phase.

## Introduction

Since the discovery of the 14π-aromatic anthracene molecule (C_14_H_10_) as a product of coal-tar distillation by Dumas and Laurent in 1832^1^, polycyclic aromatic hydrocarbons (PAHs)—hydrocarbons composed of multiple fused benzenoid rings^2,3^—have been contemplated in molecular mass growth processes in combustion systems along with cold molecular clouds, hydrocarbon-rich atmospheres of planets and their moons^4^, and circumstellar envelopes of carbon-rich Asymptotic Giant Branch stars like IRC + 10216 covering temperatures from 10 K to a few 1000 K^5–8^. In deep space, PAHs have been associated with an abiotic synthesis of biorelevant molecules crucial to the earliest forms of life^8–10^. These aromatics are omnipresent in carbonaceous chondrites such as Orgueil, Murchison, and Allende and could comprise up to 30% of the cosmic carbon budget^11^. On Earth, however, PAHs together with nanometer-sized soot particles are categorized as carcinogenic byproducts released in incomplete combustion processes of fossil fuel^12^. Hence, a fundamental understanding of the individual reaction steps is required to unravel the myriad and diverse pathways to aromatics on the microscopic level from the bottom up in extreme environments^13^. The mechanisms that lead to these PAHs can also transverse unconventional reaction pathways, and can prove a rich tapestry to map out excited state chemical dynamics as is seen here in the self-reaction of the benzyl (C_7_H_7_^•^) radical leading eventually to the formation of phenanthrene and anthracene. We report on an isomer-selective synthesis of anthracene driven by a discrete, spin-dictated mechanism on an excited state potential energy surface, hitherto not seen in radical-radical reactions.

**Fig. 1:**
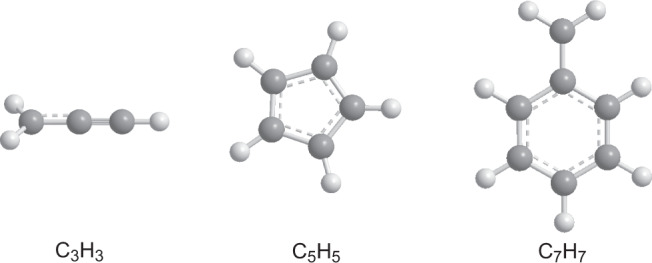
Molecular structures of three resonantly stabilized free radicals (RSFRs). Propargyl (C_3_H_3_^•^, X^2^B_2_), cyclopentadienyl (C_5_H_5_^•^, X^2^B_2_), and benzyl (C_7_H_7_^•^, X^2^B_2_) are contemplated as key precursors to polycyclic aromatic hydrocarbons (PAHs) in high temperature molecular mass growth processes. Carbon and hydrogen atoms are color coded in gray and white, respectively.

**Fig. 2:**
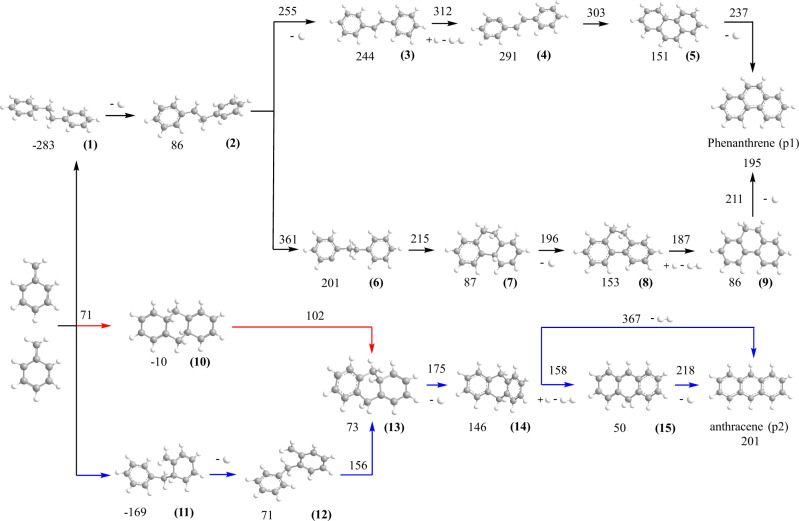
Reaction pathways for the benzyl radical self-reaction. This reaction leads to phenanthrene (p1) calculated at the CBS-QB3 level theory as extracted from Ref. ^14^. Novel reaction pathways to anthracene (p2) computed at the G3(MP2,CC)//B3LYP/6-311G(d,p) level of theory in the present work on the singlet and triplet surfaces are color coded in blue and red, respectively. All energies are presented in the unit of kJ mol^−1^. Carbon and hydrogen atoms are color coded in gray and white, respectively. Cartesian coordinates and vibrational frequencies are provided in Supplementary Table 1.

There is a paucity in molecular beam studies of the reactions between two aromatic (AR) and/or resonantly stabilized free radicals (RSFR)—organic radicals such as propargyl (C_3_H_3_^•^, X^2^B_2_), cyclopentadienyl (C_5_H_5_^•^, X^2^E_1_″), and benzyl (C_7_H_7_^•^, X^2^B_2_) with the unpaired electron delocalized over multiple carbon atoms. This is due to the previously insurmountable difficulties in preparing sufficiently high concentrations of radicals to generate detectable quantities of products (Fig. 1). The propargyl (C_3_H_3_^•^) - propargyl (C_3_H_3_^•^)^14^ and indenyl (C_9_H_7_^•^) – methyl (CH_3_^•^) systems synthesizing benzene (C_6_H_6_) and naphthalene (C_10_H_8_), respectively, have been explored in molecular beam studies^15^. Pathways to PAHs carrying multiple six-membered rings like phenanthrene (C_14_H_10_, **p1**) and anthracene (C_14_H_10_, **p2**) through the self-reaction of the 6π-aromatic *and* resonantly stabilized benzyl (C_7_H_7_^•^, X^2^B_2_) radical have been predicted theoretically (Supporting Information) (Fig. 2)^16^; however, there has been no experimental examination of this reaction leading to a simultaneous in situ detection of phenanthrene (C_14_H_10_, **p1**) *and* anthracene (C_14_H_10_, **p2**) (Supporting Information). Sinha and Raj computationally explored the benzyl radical self-reaction on the ground state singlet surface via initial recombination of the radicals with their radical centers on the singlet surface^16^. Two key pathways involve the 1,2-diphenylethane intermediate (C_6_H_5_CH_2_CH_2_C_6_H_5_, **1**), two hydrogen atom losses via *trans-*stilbene (C_14_H_12_, **3**) and 9,10-dihydrophenanthrene (C_14_H_12_, **8**), and eventually the formation of phenanthrene (C_14_H_10_, **p1**). At temperatures above 1200 K, the authors predicted that phenanthrene (C_14_H_10_, **p1**) represents the nearly exclusive product of the benzyl-radical self-reaction at levels of at least 99%. Rijs et al. exploited infrared (IR)/ultraviolet (UV) ion dip spectroscopy coupled with a high-temperature pyrolysis reactor to experimentally probe the products of the benzyl radical self-reactions at 1373 K in 1.4 bar argon buffer gas^17^, but only phenanthrene (C_14_H_10_, **p1**) was detected. Based on modeling studies of the pyrolysis of toluene, Matsugi and Miyoshi suggested that only the phenanthrene isomer might be formed via a stilbene intermediate^18^. These studies reveal that there is still limited understanding of the fundamental reaction pathways of how the benzyl radical self-reaction can lead to phenanthrene (C_14_H_10_, **p1**) and anthracene (C_14_H_10_, **p2**). Identification of both isomers would provide an experimental benchmark for the conversion of two singly ringed benzyl radicals to the 14π-aromatic system phenanthrene (C_14_H_10_, **p1**) and anthracene (C_14_H_10_, **p2**) in high-temperature combustion and circumstellar environments.

Here, we report on a combined computational and experimental study of the benzyl (C_7_H_7_^•^) radical self-reaction leading eventually to the formation of phenanthrene (C_14_H_10_, **p1**) *and* anthracene (C_14_H_10_, **p2**) as prototype 14π aromatic systems carrying three fused benzene rings. The outcome of the isomer-selective synthesis is shown to be driven by discrete, spin-dictated mechanism, with phenanthrene (C_14_H_10_, **p1**) initiated through a classical barrierless radical-radical recombination of two benzyl radicals with radical centers at the exocyclic methylene (CH_2_) moiety on the singlet ground state surface. Formation of anthracene (C_14_H_10_, **p2**) commences unconventionally on the excited state triplet surface (a^3^A) through [3 + 3] cycloaddition involving a transition state with a cyclic arrangement of the atoms in a six-membered ring along with a reorganization of σ and π bonds via excited-state dynamics initiated by a single collision. The excited-state dynamics leading eventually to anthracene (C_14_H_10_, **p2**) defy conventional wisdom that PAH formation via radical-radical reactions solely takes place on electronic ground state (singlet) surfaces via initial recombination of the doublet reactants at their radical centers. The facile formation of anthracene (C_14_H_10_, **p2**) via excited-state dynamics on the triplet surface through cycloaddition as showcased here presents a fundamental shift in currently “accepted” views and opens up the door for a more “rapid” synthesis of aromatic, multi-ringed structures such as three-ring PAHs from mono-ringed radical precursors (benzyl) at high-temperature conditions relevant to combustion and deep space. It further delivers a strategy to explore chemical reactions of aromatic radicals and RSFR under high temperature environments of relevance to synthetic and materials chemistry leading eventually to carbonaceous nanostructures like fullerenes, nanocages, and nanotubes^19–22^.

## Results & discussion

The formation of phenanthrene (C_14_H_10_, **p1**) and anthracene (C_14_H_10_, **p2**) is initiated through the reaction of the benzyl radical (C_7_H_7_^•^, X^2^B_2_) generated via pyrolysis of helium-seeded benzylbromide (C_7_H_7_Br) at fractions of 0.15% in a chemical micro reactor at temperature of 1473 ± 10 K and a reactor inlet pressure of 400 mbar^23^. Under our experimental conditions, homolytic cleavage of the carbon-bromine bond leads exclusively to atomic bromine plus a benzyl radical^24^. Although the formation of the phenanthrene (C_14_H_10_, **p1**) isomer has been predicted theoretically^16^ and demonstrated experimentally^17^, there is no experiment to date that has followed the mechanism of the benzyl radical self-reaction leading to the simultaneous gas-phase detection of phenanthrene (C_14_H_10_, **p1**) and anthracene (C_14_H_10_, **p2**) under controlled experimental conditions with tunable vacuum ultraviolet (VUV) light to interrogate the reaction products in a molecular beam. It should be noticed that when the initial reactants are dense without dilution, the consequent reactions from tricyclic PAH radicals (anthrancenyl/phenanthrenyl radicals generated from the H-abstraction of anthracene/phenanthrene) might lead to the formation of tetracyclic PAHs such as tetracene, chrysene, and pyrene^25,26^. Thus, the dilute reactant conditions at elevated temperatures, used in our method is crucial in detecting isomers and arresting reactions before larger PAHs are formed in subsequent reactions.

**Fig. 3:**
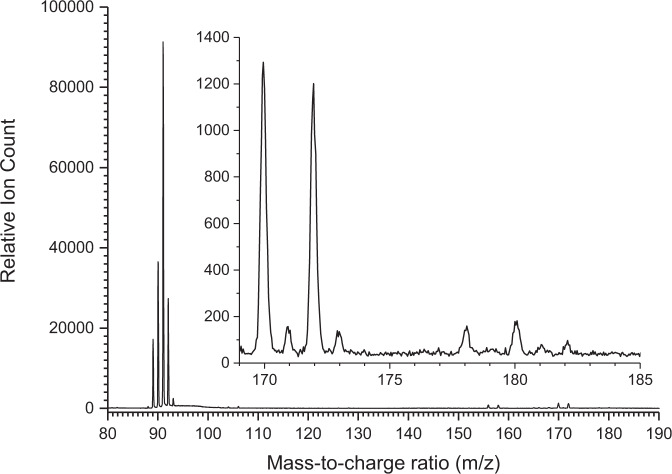
Photoionization mass spectrum. It was recorded at a photon energy of 9.50 eV for the benzyl radical self-reaction at a reactor temperature of 1473 K; the insert highlights the ion signal from m/z = 169–185.

**Fig. 4:**
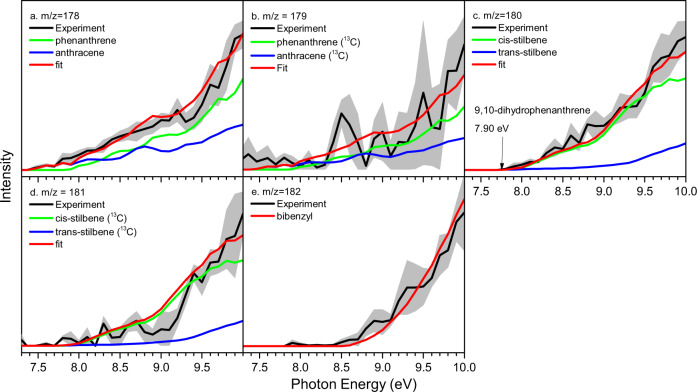
Photoionization Efficiency (PIE) Curves. PIE curves for different product masses are shown in panel (**a**) m/z = 178, (**b**) m/z = 179, (**c**) m/z = 180, (**d**) m/z = 181, and (**e**) m/z = 182. They were recorded for the benzyl radical self-reaction at a reactor temperature of 1473 ± 10 K. Black: experimental PIE curves; blue/green/red: reference PIE curves; the red line resembles the overall fit. The error bars consist of two parts: ±10% based on the accuracy of the photodiode and a 1 σ error of the PIE curve averaged over the individual scans.

A representative mass spectrum collected at a photon energy of 9.50 eV for the benzyl radical self-reaction is presented in Fig. 3 for a reactor temperature of 1473 K. Ion counts are observable up to a mass-to-charge ratio (m/z) of 182 (C_14_H_14_^+^). Formally, this m/z is twice the mass-to-charge ratio of the ionized benzyl radical (C_7_H_7_^+^) of m/z = 91. Photoionization efficiency curves (PIE), which report the intensity of a well-defined ion of a specific m/z ratio as a function of photon energy, are used to identify the structural isomer(s) formed in the benzyl self-recombination (Fig. 4). Three independent experimental measurements between 7.3 and 8.0 eV, measured in step size of 0.1 eV, with 300 Torr helium backing pressure are reported in Supplementary Data 1–3 while three independent experimental measurements between 8.0 and 10.0 eV measured in step size of 0.1 eV are reported in Supplementary Data 4–6. These PIE curves then can be fitted with a (linear combination of) reference curve(s) of distinct structural isomer(s)^27^. A close look at the PIE curve of m/z = 182 (Fig. 4) reveals that these data can be nicely replicated with the PIE reference curve of 1,2-diphenylethane (bibenzyl, C_6_H_5_CH_2_CH_2_C_6_H_5_, **1**) as the initial recombination product of two benzyl radicals. The adiabatic ionization energy (IE) of (**1**) of 8.7 ± 0.1 eV^28^ agrees well with the onset of ion counts of 8.60 ± 0.05 eV in the PIE curve. The ion signal at m/z = 180 (Fig. 3) is twice as strong as the ion counts for m/z = 182. The PIE curve at m/z = 180 (C_14_H_12_^+^, Fig. 4) cannot be replicated with a single contributor, but contributions from *trans-* and *cis-*stilbene (C_6_H_5_CH = CHC_6_H_5_, **3/3’**) at ion fractions of 22.5 ± 2.3% and 77.5 ± 7.8% at 10.00 eV are required. It is important to highlight that the PIE curves of 9,10-dihydrophenanthrene (**8**) or 9,10-dihydroanthracene (**14**) could not fit the experimental PIE curve at m/z = 180. However, minor contributions of up to 4.7 ± 0.5% (**8**) and 2.5 ± 0.3% (**14**) can be accounted for without changing the overall fit of the PIE curve at m/z = 180. Further, the ion counts at m/z = 178 are linked to a hydrocarbon with the molecular formula C_14_H_10_ (Fig. 3). A close look at the PIE curve of m/z = 178 (Fig. 4) reveals that a linear combination of PIE reference curves for phenanthrene (C_14_H_10_, **p1**) *and* anthracene (C_14_H_10_, **p2**) is critical to replicate the experimental data at m/z = 178. In detail, the experimental PIE curve at m/z = 178 shows an onset of ion counts at 7.45 ± 0.05 eV, which correlates exceptionally well with the NIST evaluated adiabatic IE of anthracene of 7.439 ± 0.006 eV. A sole contribution of anthracene, however, cannot replicate ion counts above 7.9 eV. To reproduce the overall shape of the PIE curve, a second contribution of the PIE curve of phenanthrene (IE = 7.891 ± 0.001 eV) is required. Accounting for the photoionization cross sections and quoted errors of 20%, described in the Supplementary Fig. 3, fractions of phenanthrene and anthracene of 87 ± 17% and 13 ± 3% are extracted i.e., the phenanthrene isomer dominates at m/z 178. Signal at m/z = 179 and 181 is much weaker compared to m/z = 178 and 180, respectively, and can be linked to the ^13^C analogues of phenanthrene (C_14_H_10_, **p1**) *and* anthracene (C_14_H_10_, **p2**) (m/z = 178) and *trans-* and *cis-*stilbene (C_6_H_5_CH = CHC_6_H_5_, **3/3’**) (m/z = 180) present at levels of 15.4 % accounting for naturally occurring ^13^C (1.1%) and fourteen carbon atoms in **p1, p2**, and (**3/3’**).

Besides ion counts (m/z = 178–182), the mass spectrum also shows ion counts at m/z = 170 and 172. These are linked to non-pyrolyzed C_7_H_7_^79^Br and C_7_H_7_^81^Br precursors, respectively (Supplementary Figs. 1 and 2). Further, ion counts at m/z = 91, 92, and 93 can be associated with the benzyl radical (C_7_H_7_^+^, m/z = 91), the ^13^C substituted benzyl radical (^13^CC_6_H_7_^+^) and toluene (C_6_H_5_CH_3_) (m/z = 92), and ^13^C substituted toluene (^13^CC_6_H_8_) as well as the doubly ^13^C substituted benzyl radical (^13^C_2_C_5_H_7_^+^) (m/z = 93). Control experiments of helium-seeded benzylbromide conducted under identical experimental conditions, but keeping the silicon carbide tube at 293 K, verify that the aforementioned products are not contaminations from the reactants, but clearly reaction products from the benzyl radical-self reaction. Overall, our experimental study provided compelling evidence on the identification of both phenanthrene (C_14_H_10_, **p1**) *and* anthracene (C_14_H_10_, **p2**) (178 amu) with fractions of 87 ± 17% and 13 ± 3% along with *cis/trans*-stilbene intermediates (C_14_H_12_, **3/3’**, 180 amu) and the 1,2-diphenylethane (C_14_H_14_, **1**, 182 amu) adduct along with possibly minor fractions of 9,10-dihydrophenanthrene and 9,10-dihydroanthracene (C_14_H_12_, **8/14**, 180 amu). The quantification of anthracene (C_14_H_10_, **p2**) contradicts previous electronic structure and flame modeling studies^16^ that the benzyl radical self-reaction should lead under our experimental conditions to nearly exclusive production to the phenanthrene (C_14_H_10_, **p1**) molecule with upper limits of anthracene (C_14_H_10_, **p2**) of 1% at most. These deviations by at least one order of magnitude suggest that an understanding of the critical routes to anthracene (C_14_H_10_, **p2**) are lacking.

The aforementioned discrepancies call for a computational investigation of the benzyl radical self-reaction beyond the traditional singlet ground state surface leading to phenanthrene (C_14_H_10_, **p1**)^16^. Figure 2 compiles the theoretically predicted key pathways dominating the formation of phenanthrene (C_14_H_10_, **p1**) on the electronic ground state singlet surface commencing with the radical-radical recombination through their radical centers located at the methylene (CH_2_) moiety of the benzyl radical^16^; novel pathways leading to anthracene (C_14_H_10_, **p2**) on the singlet and triplet surfaces are presented via color codes in red and blue, respectively. The traditional viewpoint of the benzyl-radical self-reaction suggests a recombination of the benzyl radical on the ground state singlet surface with their radical centers at the CH_2_ moieties leading to 1,2-diphenylethane (C_6_H_5_CH_2_CH_2_C_6_H_5_, **1**). The latter can emit a hydrogen atom from one of the CH_2_ groups leading to 1,2-diphenylethyl (C_6_H_5_CH^•^CH_2_C_6_H_5_, **2**) or might be stabilized in the reactor by a three-body reaction with the helium buffer gas. Two reaction pathways from (**2**) eventually lead via *trans-*stilbene (C_14_H_12_, **3**) or 9,10-dihydrophennthrene (C_14_H_12_, **8**) through three hydrogen atom losses along with cyclization to phenanthrene (C_14_H_10_, **p1**). The overall reaction endoergicity of 195 kJ mol^−1^ can be compensated by the elevated temperature of the reactor of 1,473 ± 10 K; the reaction progress is facilitated by hydrogen atom abstraction by hydrogen atoms present in the system, in particular for the (**3**)–(**4**), (**8**)–(**9**), and (**14**)–(**15**) steps. The energetics of the transition states suggests that formation of phenanthrene (C_14_H_10_, **p1**) via the *trans-*stilbene (C_14_H_12_, **3**) is preferred; the highest energy transition state (**3**) + H → (**4**) + H_2_ of 312 kJmol^−1^ is still below the barrier of 361 kJmol^−1^ for the (**2**) → (**6**) isomerization required for the 9,10-dihydrophenenthrene (C_14_H_12_, **8**) route. These findings are in line with the experimental observations of the 1,2-diphenylethane (C_6_H_5_CH_2_CH_2_C_6_H_5_, **1**) and *trans-*stilbene (C_14_H_12_, **3**) intermediates supporting the *trans-*stilbene (C_14_H_12_, **3**) route; however, the 1,2-diphenylethane (C_6_H_5_CH_2_CH_2_C_6_H_5_, **1**) pathway cannot be completely discounted (Fig. 4). Note that the pathway to phenanthrene (C_14_H_10_, **p1**) only proceeds via *trans-*stilbene (C_14_H_12_, **3**), but in the reactor, *trans-*stilbene (C_14_H_12_, **3**) can undergo hydrogen atom assisted isomerization to *cis-*stilbene (C_14_H_12_, **3’**)^29^.

**Fig. 5:**
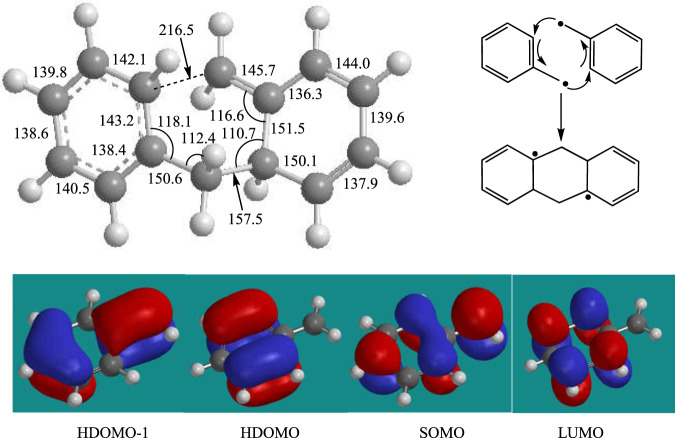
Structure and frontier orbitals involved in the benzyl radical self-reaction. Top left: Structure of the transition state of the cycloaddition reaction of two benzyl radicals to triplet 4a,8a,9,10-tetrahydroanthracene (C_14_H_14_, **10**). Bond lengths are given in pm and angles in degrees. Top right: schematics of electron redistribution in the reaction of two benzyl radicals. Bottom: frontier orbitals including the lowest unoccupied molecular orbital (LUMO), the singly occupied molecular orbital (SOMO), the highest doubly occupied molecular orbital (HDOMO) and HDOMO-1 involved in the formation of 4a,8a,9,10-tetrahydroanthracene (C_14_H_14_, **10**) from two benzyl radials via cycloaddition of two doublet radicals.

Having commented on the route to phenanthrene (C_14_H_10_, **p1**), we discuss the newly revealed reaction pathways leading to anthracene (C_14_H_10_, **p2**). These are highlighted in Fig. 2 and color-coded in red and blue for the triplet and singlet surfaces, respectively. ***First***, the benzyl radical can also add with its radical center at the CH_2_ moiety to one of the ortho-positions of the second benzyl radical on the singlet surface yielding the C_14_H_14_ intermediate (**11**); careful investigations at the B3LYP/6-311 G(d,p) level of theory by scanning the minimal potential energy reaction profile reveal that this pathway is barrierless. A subsequent hydrogen atom loss from the ortho position leads to the C_14_H_13_ intermediate (**12**), which can isomerize via ring closure to (**13**). This intermediate carries the carbon backbone of the anthracene molecule and undergoes a subsequent hydrogen atom loss yielding 9,10-dihydroanthracene (C_14_H_12_, **14**). Two additional hydrogen atom losses, where the first one, (**14**) + H → (**15**) + H_2_, actually occurs by hydrogen abstraction from (**14**) by atomic hydrogen eventually yield anthracene (C_14_H_10_, **p2**) via intermediate (**15**). A one-step molecular hydrogen loss is not competitive in the presence of a sufficient concentration of hydrogen atoms considering the inherent transition state located 367 kJmol^−1^ above the energy of the separated reactants compared to the hydrogen atom abstraction and loss transition states placed only 158 and 218 kJmol^−1^ above the separated reactants. ***Second***, both benzyl radicals can also recombine on the triplet surface (a^3^A) via *[3* + *3] cycloaddition* through a transition state in the entrance channel located 71 kJmol^−1^ above the energy of the separated reactants leading to triplet 4a,8a,9,10-tetrahydroanthracene (C_14_H_14_, **10**). This intermediate contains three rings and is formed via a single collision between two benzyl radicals via *cycloaddition*. The extensive reorganization of the σ- and π-electron densities involving the frontier π and π* orbitals of the two reacting benzyl radicals account for the ‘tight’ nature of this transition state (Fig. 5). Interestingly, (**10**) does not exist in a singlet electronic state. When optimized starting with the triplet geometry and open-shell singlet initial wavefunction, the structure undergoes spontaneous opening of the central ring and the optimization converges to the open singlet structure (**11**). On the triplet surface, after being produced, (**10**) emits a hydrogen atom to form intermediate (**13**), which eventually loses two hydrogen atoms yielding anthracene (C_14_H_10_, **p2**) as detected experimentally (Figs. 3 and 4). It is important to comment on the entrance channels leading to (**10**) and (**11**) on the triplet and singlet surface, respectively. Both pathways (**10**) → (**13**) and (**11**) → (**12**) + H → (**13**) + H eventually lead via (**13**) to 9,10-dihydroanthracene (C_14_H_12_, **14**) and anthracene (C_14_H_10_, **p2**). Although the formation of (**10**) on the triplet surface has the entrance and (**10**) → (**11**) + H barriers of 71 and 102 kJmol^−1^, respectively, and, hence, appears to be unfavorable compared to the barrierless path to (**11**) on the singlet surface, isomerization of (**12**)–(**13**) is not efficient with a barrier of 156 kJmol^−1^. Consequently, the pathway on the triplet surface (**10**) → (**13**) wins over the reaction sequence (**11**) → (**12**) + H → (**13**) + H on the singlet surface. This is due to the unfavorable barrier to isomerization of (**12**) to (**13**) that is twice compared to the energy of the transition state leading to (**10**) on the triplet surface. The highest barrier to anthracene (C_14_H_10_, **p2**) formation of 218 kJmol^−1^ connects to the atomic hydrogen loss of (**15**) to form the final anthracene product. This barrier is substantially lower than the barrier of 312 kJmol^−1^ for the hydrogen abstraction by atomic hydrogen from (**3**) forming (**4**) in the most favorable pathway to phenanthrene (C_14_H_10_, **p1**) via (**1**) → (**2**) + H → (**3**) + 2 H → (**4**) + H + H_2_ → (**5**) + H + H_2_ → **p1** + 2 H + H_2_. Therefore, anthracene (C_14_H_10_, **p2**) formation can compete with the synthesis of phenanthrene (C_14_H_10_, **p1**). The reactivity is also influenced by the cone-of-acceptance of the benzyl radical leading to the initial collision complexes (**1**) [forming phenanthrene] versus (**10**) [forming anthracene] on the singlet and triplet surface, respectively. While the formation of (**10**) is geometrically constrained due to the cycloaddition character of the transition state (Fig. 5) and dictated by low impact parameters, the preferred synthesis of phenanthrene (C_14_H_10_, **p1**) over anthracene (C_14_H_10_, **p2**) with branching ratios of 87 ± 17% and 13 ± 3%, respectively, suggests that (**1**) is accessible through a larger range of impact parameters.

The mechanisms to form both phenanthrene and anthracene beginning from C_7_H_7_ + C_7_H_7_ are very complex as they involve series of consecutive reactions: C_7_H_7_ + C_7_H_7_ → C_14_H_13_ (**13**) + H, (**13**) → C_14_H_12_ (**14**) + H, (**14**) + H → C_14_H_11_ (**15**) + H_2_, (**15**) → C_14_H_10_ (**p2**) + H for anthracene, and C_7_H_7_ + C_7_H_7_ → C_14_H_14_ (**1**)/C_14_H_13_ (**2**) + H, [C_14_H_14_ (**1**) + H → C_14_H_13_ (**2**) + H_2_], C_14_H_13_ (**2**) → C_14_H_12_ (**3**) + H, C_14_H_12_ (**3**) + H → C_14_H_11_ (**4**) + H_2_, (**4**) → C_14_H_10_ (**p1**) + H for phenanthrene; the reaction system for phenanthrene is even much more complicated^16,18^ than the snapshot provided here. Therefore, the relative yields of phenanthrene and anthracene are controlled by multiple factors including rate constants of all reactions involved under particular conditions and concentrations of atomic and molecular hydrogen, which can be generated or consumed by competing reactions occurring in the system. Detailed modeling of the phenanthrene to anthracene branching ratio would be specific for particular flame conditions and is beyond of the scope of the present study. The existing models^16,18^ can be extended by rate constants for the reactions along the anthracene pathway initiating on the triplet surface which are provided in the Supporting Information (Supplementary Table 1). In the entrance channel on the phenanthrene pathway, the recombination of two benzyl radicals proceeds by the well-skipping C_7_H_7_ + C_7_H_7_ → C_14_H_13_ (**2**) + H mechanism because, if collisionally stabilized C_14_H_14_ (**1**) is formed, it is unlikely to unimolecularly decompose to C_14_H_13_ (**2**) + H as the dissociation pathway back to C_7_H_7_ + C_7_H_7_ is more favorable and the rate constant for the forward decomposition is very low^16,18^. Therefore, we compare the rate constant of the triplet reaction C_7_H_7_ + C_7_H_7_ → C_14_H_13_ (**13**) + H with that for C_7_H_7_ + C_7_H_7_ → C_14_H_13_ (**2**) + H^18^ (Supplementary Fig. 4(a)). At 1500 K the rate constant for the singlet channel is higher than that for the triplet channel. Among the consequent reaction steps, the hydrogen atom loss reactions from C_14_H_13_ and C_14_H_11_ are very fast at relevant temperatures (Supplementary Fig. 4(b)); in fact, the present pressure-dependent calculations predict that the radicals are unstable at temperatures and pressures typical for the micro reactor and immediately equilibrate with their H loss products. However, the rate constants for hydrogen abstraction by atomic hydrogen are significantly higher for the anthracene pathway; for instance, at 1500 K the rate constant for (**14**) + H → (**15**) + H_2_ is a factor of more than 30 higher than that for (**3**) + H → (**4**) + H_2_. Thus, the reactions following the formation of (**13**) in the anthracene channel are noticeably faster than those following the formation of (**2**) in the phenanthrene channels, which results in an increase of the relative yield of anthracene. However, it is not possible to predict the phenanthrene to anthracene branching ratio without detailed modeling taking into account the distribution of temperature and pressure in the reactor as well as the concentrations of atomic hydrogen and alternative abstractors like bromine atoms, which affect the rates of bimolecular reactions participating in the network.

To sum up, our combined experimental and computational study identified phenanthrene (C_14_H_10_, **p1**) and anthracene (C_14_H_10_, **p2**) as two prototype 14π aromatic products of the benzyl radical self-reaction at elevated temperatures of 1473 ± 10 K representing high-temperature combustion systems and carbon-rich circumstellar envelopes of, e.g., IRC + 10216 star. The isomer-selective synthesis is driven by two highly diverse reaction mechanisms. A radical-radical recombination of two benzyl radicals with radical centers at the methylene (CH_2_) moiety leads initially to 1,2-diphenylethane (C_6_H_5_CH_2_CH_2_C_6_H_5_, **1**) followed by hydrogen losses and ring closure to eventually phenanthrene (C_14_H_10_, **p1**). The formation of anthracene (C_14_H_10_, **p2**) embarks preferentially on the excited state triplet surface (a^3^A) through [3 + 3] cycloaddition via a transition state with a cyclic arrangement of the atoms in a six-membered ring together with an extensive reorganization of σ and π bonds via excited state dynamics initiated by a single collision between two radicals. These excited state dynamics producing eventually to anthracene (C_14_H_10_, **p2**) challenge ‘established’ paradigms that PAH formation via radical-radical reactions solely operates on electronic ground state (singlet) surfaces through recombination of the doublet reactants at their radical centers. It should be noted that excited state dynamics are of fundamental importance in polymer chemistry, too. Here, polymerization mechanisms involving excited state anions have been identified as elementary reaction pathways in an anionic isoprene polymerization implicating electronic excitation of a polyisoprene-isoprene complex to a quasi-degenerate electronically excited state^30^. Recently, Rodembusch et al.^31^ synthesized eight fluorescent monomers with emissions at long wavelengths originating from an excited keto tautomer; the latter arises from an enol-cis conformer in the electronically excited state. Thus, the facile formation of anthracene (C_14_H_10_, **p2**) via excited-state dynamics on the triplet surface through cycloaddition involving two doublet radicals represents a fundamental shift in currently “perceived” views toward the synthesis of multi-ringed structures in the gas phase broadening our understanding of the origin and evolution of carbonaceous matter in the Universe.

## Methods

### Experimental

The reaction between two benzyl radicals (C_7_H_7_) was examined under combustion-relevant conditions by utilizing a resistively heated high-temperature pyrolytic reactor^23^ incorporated into a molecular-beam chamber equipped with a Wiley–McLaren Reflectron time-of-flight mass spectrometer (Re-TOF) at the Advanced Light Source (ALS) at the Chemical Dynamics Beamline^14^. Briefly, a continuous beam of benzyl radicals (C_7_H_7_) was generated in situ through the pyrolysis of benzylbromide (C_7_H_7_Br) (Sigma Aldrich, 98%) at 1473 K via carbon-bromine bond cleavage at concentrations of 0.15% in helium carrier gas at total pressures of 400 mbar at the reactor inlet^24^. At 298 K, the vapor pressure of benzylbromide is 0.6 mbar. Upon exiting the heated silicon carbide (20 mm) tube and passing through a skimmer, the neutral molecules within the supersonic beam were photoionized by single-photon ionization utilizing quasi-continuous tunable VUV radiation. A mass spectrum was obtained at intervals of 0.05 eV between 7.30 and 10.00 eV. The Re-TOF spectrometer was operated with 2.5 µs repeller pulses. Photoionization efficiency curves (PIEs), which report the ion counts at a particular mass-to-charge (m/z) ratio as a function of photon energy, were obtained by integrating the signal at a well-defined m/z ratio selected for the species of interest over the energy range and normalizing to the total photon flux. Note that whenever necessary, calibration PIE curves were recorded within the same experimental setup.

### Computational

Earlier computational investigations of the benzyl radical self-reaction have been limited to their recombination at the radical center^16^. A potentially important pathway via the ‘head-tail’ recombination has been not explored to date. Here, ab initio G3(MP2,CC)//B3LYP/6-311G(d,p) calculations have been employed to explore the lowest singlet and triplet C_14_H_14_ PESs accessed by the reaction of the benzyl radical with its CH_2_ group attacking the ortho position in the ring of its counterpart. The Cartesian coordinates (in Å) and vibrational frequencies (in cm^−1^) for reactants, intermediates, transition states, and products along reaction pathways leading to phenanthrene and anthracene are reported in Supplementary Data 7. Then, the C_14_H_x_ (x = 13–10) species formed by consequent H losses from C_14_H_14_ in their ground doublet and singlet electronic states were also explored at the same level of theory. The G3(MP2,CC)//B3LYP/6-311G(d,p) model chemistry scheme^32–34^, which is expected to provide accuracy of 4–8 kJ mol^−1^ for relative energies, involves geometry optimization and vibrational frequencies calculations at the hybrid density functional B3LYP/6-311G(d,p) level^35,36^ followed by single-point energy calculations at the CCSD(T)/6-311 G(d,p), MP2/6-311 G(d,p), and MP2(G3Large) levels of theory aimed to evaluate the CCSD(T) energy with a large and flexible G3Large basis set. The overall energy in this scheme also includes zero-point vibrational energy corrections ZPE obtained at the B3LYP/6-311 G(d,p) level. Connections between transition states and local minima they link were verified by intrinsic reaction coordinate (IRC) calculations. The family of the G2–G4 model chemistry schemes has been shown^32–34,37^ to consistently achieve similar accuracy not only for closed shell singlet molecules but also for radicals (doublets) and diradicals (triplets) when treating the lowest state for each particular spin with a predominantly single-reference character of the wavefunction. The absence of a significant multireference character in the wavefunctions of all species considered in the present study is indicated by low values of their T1 diagnostics^38,39^. Therefore, we anticipate that the accuracy of the calculated energies on the singlet, triplet, and doublets PESs are comparable here. The ab initio calculations were performed utilizing the Gaussian 09^40^ and MOLPRO 2015^41^ program packages. Temperature- and pressure-dependent phenomenological rate constants for the reactions on the anthracene pathway were computed using the one-dimensional Rice–Ramsperger–Kassel–Marcus-Master Equation (RRKM-ME) approach^42^ employing the MESS software package^43^ within the rigid rotor-harmonic oscillator approximation for partition function calculations, which utilized the G3(MP2,CC) relative energies and B3LYP/6-311G(d,p) molecular parameters.

## Supplementary information

Supplementary Information

Description of Additional Supplementary Files

Supplementary Data 1

Supplementary Data 2

Supplementary Data 3

Supplementary Data 4

Supplementary Data 5

Supplementary Data 6

Supplementary Data 7

## Data Availability

The data that support the plots within this paper and other finding of this study are available from the corresponding author upon reasonable request. The raw data (Time-of-Flight Mass Spectra as a function of photon energy) generated in this study are provided as Supplementary Data 1–6.
