# Supplementary material for: Unconventional excited-state dynamics in the concerted benzyl (C7H7) radical self-reaction to anthracene (C14H10)
Source: Nat Commun. 2022 Feb 10;13:786. doi: 10.1038/s41467-022-28466-7 (PMC8831467; doi:10.1038/s41467-022-28466-7)
Supplement: Supplementary file 1 — Supplementary Information [file 41467_2022_28466_MOESM1_ESM.pdf]

Supplementary Information

Unconventional Excited-State Dynamics in the Concerted Benzyl (C_7H_7) Radical Self-Reaction to Anthracene ($C_{14}H_{10}$)

R.I. Kaiser et al.

Supplementary Note 1

Literature survey of the Benzyl Radical Self-Reaction

Below we describe in detail what is known on the benzyl-benzyl radical-radical reaction to date. Troe et al. experimentally derived rate constants of the benzyl-benzyl reaction in the temperature range from 900 K to 1500 K of 6.6×10^{-12} $(T/1000)^{0.4}$ cm^3 s^{-1} in the presence of argon buffer gas. These data suggest a barrierless recombination of two benzyl radicals at their radical centers located at the CH_2 moieties yielding the 1,2-biphenylethane ($C_6H_5CH_2CH_2C_6H_5$) molecule.[1] Lesclaux reported rate constants of the benzyl radical self-reaction of $2.9 \pm 0.3 \times 10^{-11}$ cm^3 s^{-1} from 435 K to 519 K,[2] i.e. a factor of 5 to 6 faster than Troe et al.'s shock wave data. These studies reveal limiting high-pressure rate constants of $4.1 \pm 0.3 \times 10^{-11}$ $(T/300)^{0.23}$ cm^3 s^{-1} at pressures below 1 bar.[3] This translates to a rate of 4.4×10^{-11} cm^3 s^{-1} at 400 K, which agrees exceptionally well with Lesclaux et al.'s data. Recent cavity ring down spectroscopy of the benzyl radical self-reaction reported rates of $1.29 \times 10^{-3} \times T^{-2.669} \times e^{(-674/T)}$ cm^3 s^{-1} from 295 K to 800 K;[4] this results in reaction rates of 2.7×10^{-11} cm^3 s^{-3}, which also agrees nicely with Troe et al.'s and Lesclaux et al.'s works.

However, despite the converging data on the rate constants, there is still a limited understanding of the fundamental reaction pathways of how the benzyl radical self-reaction can lead to anthracene and/or phenanthrene. Sinha and Raj explored the reaction mechanisms of two benzyl radicals initiated through the exoergic (-283 $kJmol^{-1}$) formation of 1,2-biphenylethane ($C_6H_5CH_2CH_2C_6H_5$) computationally.[5] This study predicted multiple reaction pathways to anthracene and phenanthrene via hydrogen abstraction from the phenyl ring and/or the CH_2 moieties at the aliphatic chain of the 1,2-biphenylethane ($C_6H_5CH_2CH_2C_6H_5$) molecule followed by extensive isomerization through, e.g. ring closure) and successive hydrogen abstraction pathways. At temperatures above 1,200 K, the authors predicted that phenanthrene ($C_{14}H_{10}$) represents the main product of the benzyl-radical self-reaction. As the temperature rises to 2,000 K, anthracene was predicted to be formed at levels of a few percent compared to phenanthrene. Rijs et al. exploited infrared (IR) / ultraviolet (UV) ion dip spectroscopy coupled with a high-temperature pyrolysis reactor to experimentally probe the products of the benzyl radical self- reactions at 1,373 K in 1.4 bar argon buffer gas.[6] The authors proposed that the detected phenanthrene ($C_{14}H_{10}$) forms via a 1,2-biphenylethane ($C_6H_5CH_2CH_2C_6H_5$) intermediate, which eventually undergoes cyclization accompanied by hydrogen loss to 9,10-dihydrophenanthrene ($C_{14}H_{10}$) followed by aromatization and yet an additional molecular hydrogen loss to phenanthrene. However, the elevated pressures of the buffer

gas (1.4 bar) and the pyrolysis regime of the heated silicon carbide reactor tube of 38 mm facilitate consecutive reaction and higher-order reactions as evident from the detection of, e.g., diphenylmethane ($C_6H_5CH_2C_6H_5$) and fluorene ($C_{13}H_{10}$).

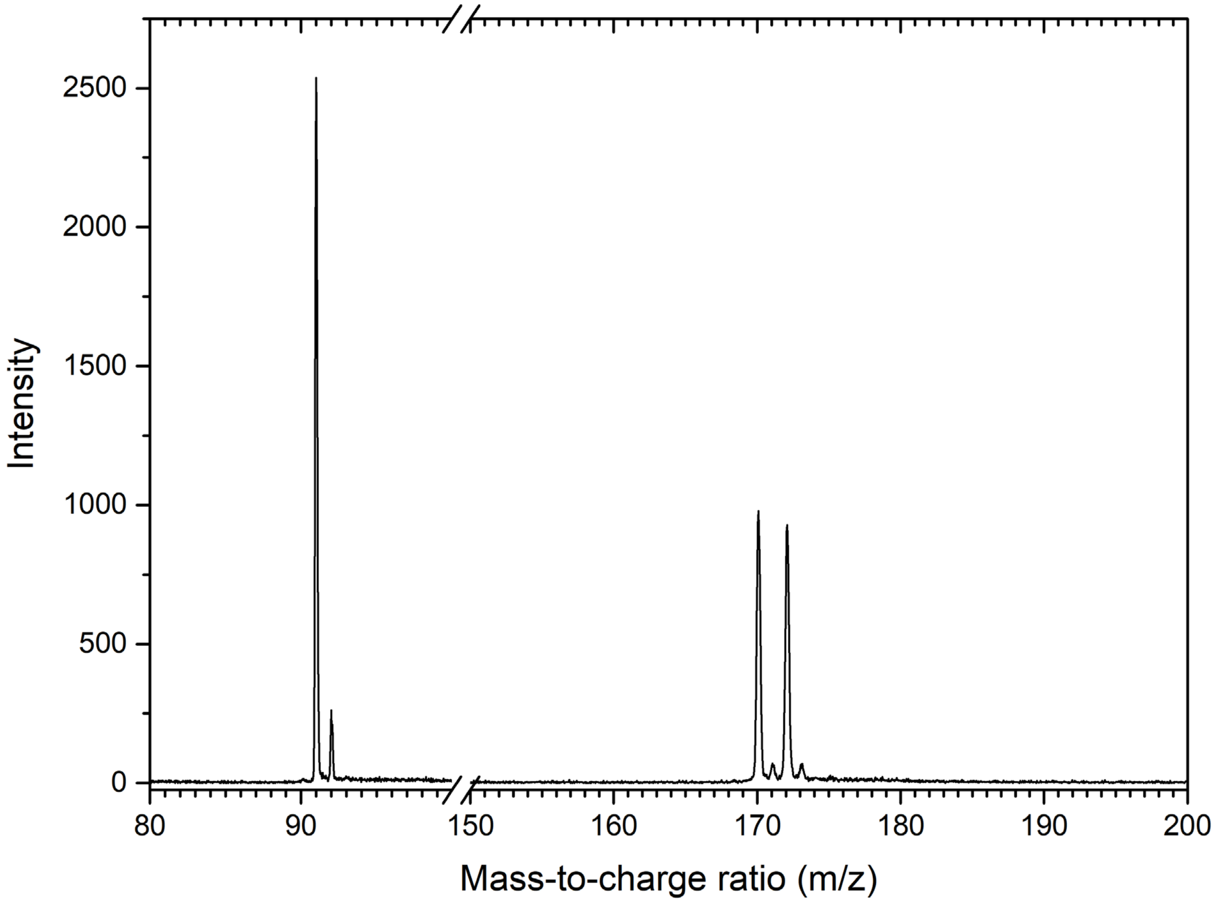

Supplementary Figure 1. Photoionization mass spectrum of helium-seeded benzyl bromide with the reactor at room temperature of 293 K. Signal at m/z = 170 and 172 is linked to the $C_7H_7{}^{79}Br$ and $C_7H_7{}^{81}Br$ precursor, respectively; signal at m/z = 171 and 173 is connected to $^{13}CC_6H_7{}^{79}Br$ and $^{13}CC_6H_7{}^{81}Br$, respectively. Signal at m/z = 91 and 92 are photofragments of the precursor yielding $C_7H_7^+$ and $^{13}CC_6H_7^+$, respectively.

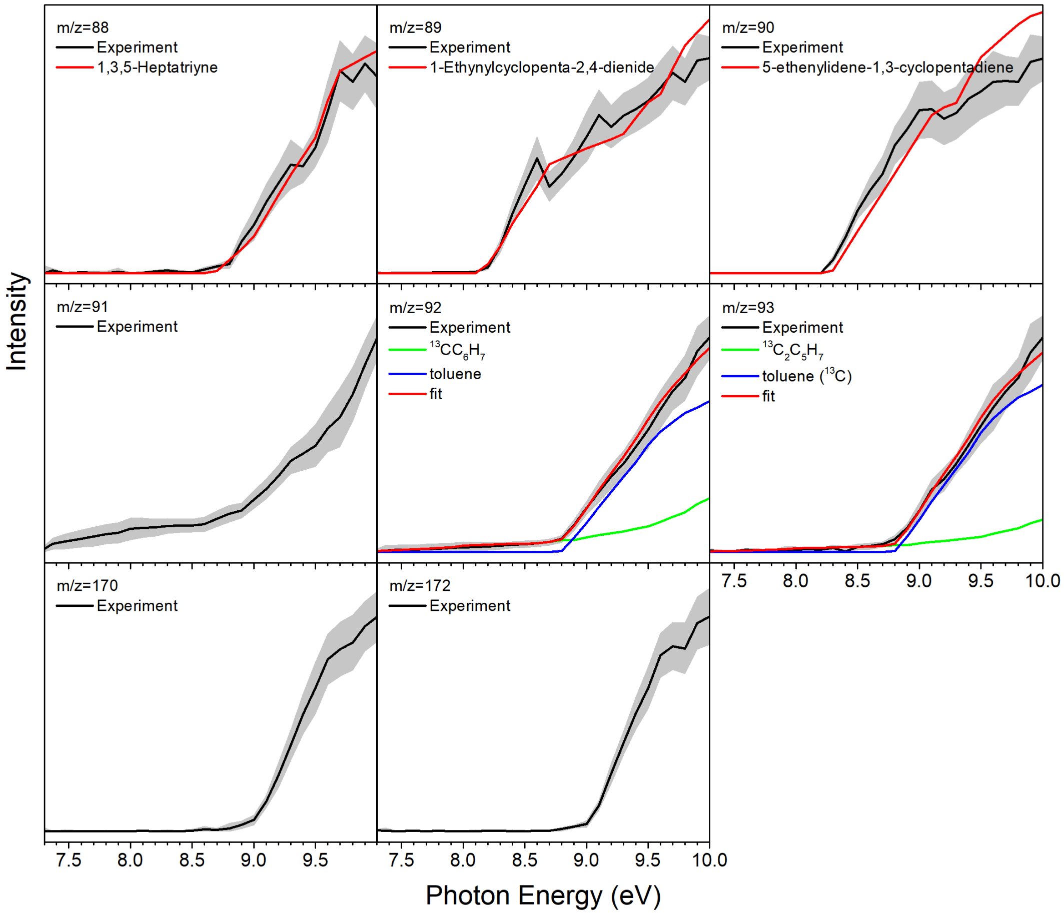

Supplementary Figure 2. PIE curves along with the fits for additional species in the benzyl (C_7H_7) recombination system. Signal at m/z = 91 can be linked to the benzyl radical (C_7H_7) prepared via the pyrolysis of bromobenzyl (C_7H_7Br, m/z = 170 and 172). The H-loss (90 amu) and H addition products (92 amu) to the benzyl radical can be also detected via signal at m/z = 90 (5-ethenylidene-1,3-cyclopentadiene, fulvenallene) and 92 (toluene C_7H_8; $^{13}CC_6H_8$, m/z = 93).[7] Further hydrogen abstraction accompanied by ring opening leads to 1,3,5-heptatriyne (m/z = 88) and 1-ethynyl-cyclopenta-2,4-dienidenyl (m/z = 89).

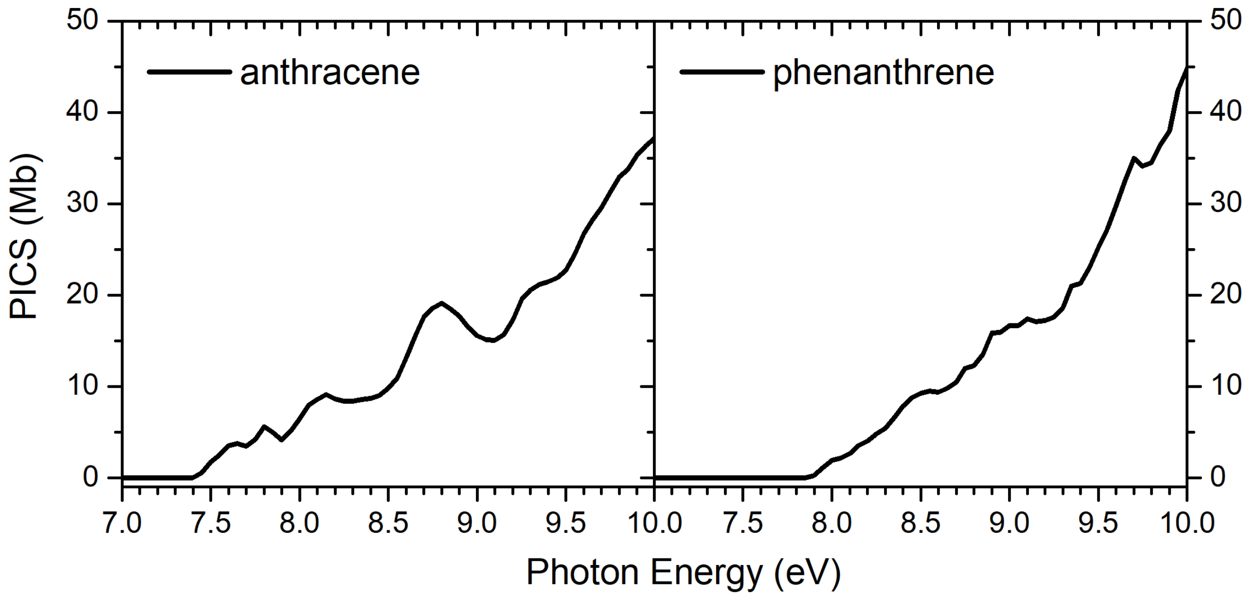

Supplementary Figure 3. Photoionization cross sections of anthracene and phenanthrene used in this work. The PIE curves of phenanthrene and anthracene were recorded in our previous published work.[8] The photoionization cross sections (PICSs) were determined from the *Photoionization Cross Section Database* database,[9] with a uncertainty of 20 %.

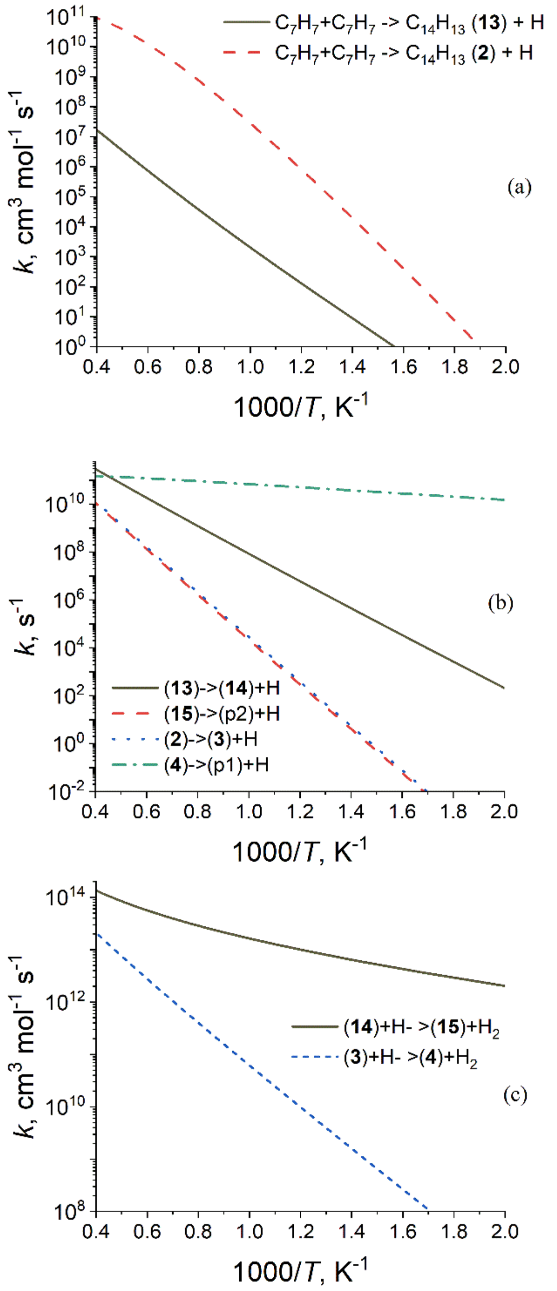

Supplementary Figure 4. Rate constants for the anthracene pathway calculated using the RRKM-ME approach in comparison with rate constants for the phenanthrene pathway taken from the literature. (a) The entrance C_7H_7 + C_7H_7 channel. The rate constant on the triplet PES is nearly independent of pressure. The rate constant on the singlet PES is computed in Ref. 18 for the pressure of 10 Torr. (b) H loss reactions from the $C_{14}H_{13}$ and $C_{14}H_{11}$ radicals. Since pressure-dependent calculations show these radicals to be unstable at relevant temperatures and pressures, high-pressure limit rate constants are shown. Rate constants for (**2**) $\rightarrow$ (**3**) + H and (**4**) $\rightarrow$ (p1) + H are taken from Ref. 16. (c) Pressure-independent rate constants of H abstraction reactions by H atoms. The rate constant for (**3**) + H $\rightarrow$ (**4**) + H_2 is taken from Ref. 16.

Supplementary Table 1: Parameters of fitted modified Arrhenius expressions $k = A\ T^{\alpha} \exp(-E_a/RT)$ within T = 500-2500 K range for the reactions on the anthracene pathway. Pre-exponential factors A are in $cm^3\ mol^{-1}\ s^{-1}$ and s^{-1} for bimolecular and unimolecular reactions, respectively, and E_a are in $cal\ mol^{-1}$.

Reaction	A	α	E_a
$C_7H_7 + C_7H_7 \rightarrow C_{14}H_{13}$ (**13**) + H[a]	5.36E+05	1.1218	26518
$C_{14}H_{13}$ (**13**) + H $\rightarrow C_7H_7 + C_7H_7$[a]	4.40E+13	-0.18553	8760.2
(**13**) $\rightarrow C_{14}H_{12}$ (**14**) + H[b]	7.79E+10	0.80419	24596
$C_{14}H_{12}$ (**14**) + H $\rightarrow$ (**13**)[b]	3.95E+08	1.577	5324.2
(**14**) + H $\rightarrow C_{14}H_{11}$ (**15**) + H_2[c]	13516	2.4296	22814
$C_{14}H_{11}$ (**15**) + $H_2 \rightarrow$ (**14**) + H[c]	2.67E+08	1.7238	1767.5
(**15**) $\rightarrow C_{14}H_{10}$ (p2) + H[b]	5.95E+10	0.84825	41089
$C_{14}H_{10}$ (p2) + H $\rightarrow$ (**15**)[b]	1.88E+09	1.5112	3444.2

[a]The rate constant was computed using the pressure dependent RRKM-ME approach but appeared to be nearly independent of pressure at relevant temperatures (T = 800-2500 K) and pressures.
[b]The high-pressure limit rate constant.
[c]The rate constant is independent of pressure.

Supplementary References

1. Brouwer, L.; Müller-Markgraf, W.; Troe, J., Thermal decomposition of toluene: a comparison of thermal and laser-photochemical activation experiments. *J. Phys. Chem.* **1988,** *92*, 4905-4914.

2. Boyd, A.; Noziere, B.; Lesclaux, R., Kinetics and thermochemistry of the reversible combination reactions of the allyl and benzyl radicals with NO. *J. Phys. Chem.* **1995,** *99*, 10815-10823.

3. Luther, K.; Oum, K.; Sekiguchi, K.; Troe, J., Recombination of benzyl radicals: dependence on the bath gas, temperature, and pressure. *Phys. Chem. Chem. Phys.* **2004,** *6*, 4133-4141.

4. Matsugi, A.; Miyoshi, A., Kinetics of the self-reactions of benzyl and o-xylyl radicals studied by cavity ring-down spectroscopy. *Chem. Phys. Lett.* **2012,** *521*, 26-30.

5. Sinha, S.; Raj, A., Polycyclic Aromatic Hydrocarbon (PAH) Formation from Benzyl Radicals: A Reaction Kinetics Study. *Phys. Chem. Chem. Phys.* **2016,** *18*, 8120-8131.

6. Hirsch, F.; Constantinidis, P.; Fischer, I.; Bakels, S.; Rijs, A. M., Dimerization of the Benzyl Radical in a High - Temperature Pyrolysis Reactor Investigated by IR/UV Ion Dip Spectroscopy. *Chem. Eur. J.* **2018,** *24*, 7647-7652.

7. Buckingham, G. T.; Porterfield, J. P.; Kostko, O.; Troy, T. P.; Ahmed, M.; Robichaud, D. J.; Nimlos, M. R.; Daily, J. W.; Ellison, G. B., The thermal decomposition of the benzyl radical in a heated micro-reactor. II. Pyrolysis of the tropyl radical. *J. Chem. Phys.* **2016,** *145*, 014305.

8. Zhao, L.; Kaiser, R. I.; Xu, B.; Ablikim, U.; Ahmed, M.; Evseev, M. M.; Bashkirov, E. K.; Azyazov, V. N.; Mebel, A. M., Low-temperature formation of polycyclic aromatic hydrocarbons in Titan's atmosphere. *Nat. Astron.* **2018,** *2*, 973-979.

9. *Photoionization Cross Section Database (Version 2.0), National Synchrotron Radiation Laboratory, Hefei, China, http://flame.nsrl.ustc.edu.cn/database/ (2017).*
