# Supplementary material for: Unconventional excited-state dynamics in the concerted benzyl (C7H7) radical self-reaction to anthracene (C14H10)
Source: Nat Commun. 2022 Feb 10;13:786. doi: 10.1038/s41467-022-28466-7 (PMC8831467; doi:10.1038/s41467-022-28466-7)
Supplement: Supplementary file 2 — Description of Additional Supplementary Files [file 41467_2022_28466_MOESM2_ESM.docx]

**Description of Additional Supplementary Files**

File Name: Supplementary Data 1
Description: Time of Flight Mass Spectra recorded between 7.3-8.0 eV with 300 Torr Helium backing pressure and MgF2 window in path of synchrotron beam of the C7H7 self reaction.

File Name: Supplementary Data 2
Description: Time of Flight Mass Spectra recorded between 7.3-8.0 eV with 300 Torr Helium backing pressure and MgF2 window in path of synchrotron beam of the C7H7 self reaction.

File Name: Supplementary Data 3
Description: Time of Flight Mass Spectra recorded between 7.3-8.0 eV with 300 Torr Helium backing pressure and MgF2 window in path of synchrotron beam of the C7H7 self reaction.

File Name: Supplementary Data 4
Description: Time of Flight Mass Spectra recorded between 8.0-10.0 eV with 300 Torr Helium backing pressure of the C7H7 self reaction.

File Name: Supplementary Data 5
Description: Time of Flight Mass Spectra recorded between 8.0-10.0 eV with 300 Torr Helium backing pressure of the C7H7 self reaction.

File Name: Supplementary Data 6
Description: Time of Flight Mass Spectra recorded between 8.0-10.0 eV with 300 Torr Helium backing pressure of the C7H7 self reaction.

File Name: Supplementary Data 7
Description: Cartesian coordinates (in Å) and vibrational frequencies (in cm-1) for reactants, intermediates, transition states, and products along reaction pathways leading to phenanthrene and anthracene shown in Figure 1.
